# The Creation of a Multidomain Neighborhood Environmental Vulnerability Index Across New York City

**DOI:** 10.1007/s11524-023-00766-3

**Published:** 2023-08-18

**Authors:** Stephen P. Uong, Jiayi Zhou, Stephanie Lovinsky-Desir, Sandra S. Albrecht, Alexander Azan, Earle C. Chambers, Perry E. Sheffield, Azure Thompson, Joseph Wilson, Jennifer Woo Baidal, Jeanette A. Stingone

**Affiliations:** 1 Department of Epidemiology, Columbia University Mailman School of Public Health, 722 West 168th Street, Room 1608, New York, NY 10032 USA; 2 Department of Pediatrics New York, Columbia University Vagelos College of Physicians and Surgeons, New York, NY USA; 3 Department of Population Health, NYU Grossman School of Medicine, New York, NY USA; 4 Department of Family and Social Medicine Bronx, Albert Einstein College of Medicine, New York, NY USA; 5 Department of Environmental Medicine and Public Health New York, Icahn School of Medicine at Mount Sinai, New York, NY USA; 6 School of Public Health Brooklyn, SUNY Downstate Health Sciences University New York, New York, NY USA

**Keywords:** Area-level vulnerability, Data integration, Environmental justice, Social determinants of health, Deprivation

## Abstract

Compared to previous studies commonly using a single summary score, we aimed to construct a multidomain neighborhood environmental vulnerability index (NEVI) to characterize the magnitude and variability of area-level factors with the potential to modify the association between environmental pollutants and health effects. Using the Toxicological Prioritization Index framework and data from the 2015–2019 U.S. Census American Community Survey and the 2020 CDC PLACES Project, we quantified census tract-level vulnerability overall and in 4 primary domains (demographic, economic, residential, and health status), 24 subdomains, and 54 distinct area-level features for New York City (NYC). Overall and domain-specific indices were calculated by summing standardized feature values within the subdomains and then aggregating and weighting based on the number of features within each subdomain within equally-weighted primary domains. In citywide comparisons, NEVI was correlated with multiple existing indices, including the Neighborhood Deprivation Index (r = 0.91) and Social Vulnerability Index (r = 0.87) but provided additional information on features contributing to vulnerability. Vulnerability varied spatially across NYC, and hierarchical cluster analysis using subdomain scores revealed six patterns of vulnerability across domains: 1) low in all, 2) primarily low except residential, 3) medium in all, 4) high demographic, economic, and residential 5) high economic, residential, and health status, and 6) high demographic, economic and health status. Created using methods that offer flexibility for theory-based construction, NEVI provided detailed vulnerability metrics across domains that can inform targeted research and public health interventions aimed at reducing the health impacts from environmental exposures across urban centers.

**Supplementary Information:**

The online version contains supplementary material available at 10.1007/s11524-023-00766-3.

## Introduction

Previous research has linked environmental pollutant exposure, including air pollution, toxic metals, and plastics, to cancer, neurodevelopmental impacts, asthma, cardiovascular disease, and other health outcomes across various urban settings [1–4]. Prior studies have conceptualized neighborhood-level features, such as housing characteristics, concentrated poverty, and health care access, as confounders or effect modifiers in evaluating the relationship between environmental pollutants and various health outcomes [5–8]. In these studies, “neighborhoods” are spatial units where individuals reside that aim to capture unique group-level properties [9]. These studies demonstrated neighborhood-level factors can contribute to environmental vulnerability differences, including greater likelihood of pollutant exposure and individual susceptibility to environmental pollutant-related health effects.

Composite indices previously used to describe neighborhood-level social and structural vulnerabilities often measure only one vulnerability domain, lack an environmental vulnerability focus, or lack options for adaptable, theory-based construction. For example, previous environmental epidemiology studies have applied the neighborhood deprivation index (NDI), calculated using purely data-driven methods, to obtain a single measure of neighborhood socioeconomic deprivation [10–14]. Similarly, the Centers for Disease Control and Prevention’s Social Vulnerability Index (SVI) summarizes several features in a single measure to reflect neighborhood-level stressors relevant to disasters or disease outbreaks [15]. Newer, neighborhood-level vulnerability indices that account for climate and environmental exposures, such as the Climate and Economic Justice Tool, often still emphasize a single summary metric and provide limited ways to compare the relative magnitude or importance of individual features or domains contributing to neighborhood vulnerability [16]. Information about specific neighborhood-level features or domains, such as the location and type of characteristic contributing to vulnerability, provides a more detailed understanding of vulnerability drivers that could inform targeted urban land use policy and environmental justice efforts. Furthermore, indices like the NDI and SVI do not include other features that may increase environmental vulnerability, including pre-existing health conditions and behaviors that may increase vulnerability to health impacts from air pollution, heat stress, and other climate stressors [10, 17–20].

Importantly, many of these indices have included neighborhood-level composition of racial/ethnic identities as a vulnerability component, although the measure does not contribute to environmental vulnerability but is rather is a proxy for structural racist policy exposures that have led to segregation, concentrated poverty, and other downstream outcomes that can increase vulnerability [21, 22]. Composite indices designed to understand the social and structural drivers of environmental vulnerability should measure the specific downstream effects of racism, rather than racial/ethnic identity as a proxy. Finally, vulnerability indices, such as the SVI and the EPA/CDC Environmental Justice Index, disaggregate vulnerability across domains but do not provide a framework for users to apply such indices for their specific purpose, limiting their ability to incorporate theory-based construction and application [23, 24].

Previous research has used the Toxicological Prioritization Index (ToxPi) [25] approach to combine multiple geospatial features into vulnerability indices across various spatial scales. In contrast to purely data-driven approaches commonly used in the construction of existing neighborhood-level indices, [10, 14, 26] ToxPi allows researchers to combine features to address research questions specific to the hyper-local needs of a given community. In this way, ToxPi allows for the characterization of vulnerability contributions across distinct domains, providing an ability to investigate whether neighborhoods may be experiencing different vulnerability contributors, thus enabling tailored intervention approaches. For example, Bhandari et al. applied ToxPi to the Houston-Galveston-Brazoria region to create a five-domain index to assist communities in developing plans to address health effects from natural disasters and industrial activity [27]. The National Institute of Environmental Health Science used a similar framework when constructing their COVID Vulnerability Index, to visualize national COVID-19 vulnerability [28], again selecting domains based on their specific goals. Both prior approaches included environmental pollution as a vulnerability component in their indices. Although this approach may be pertinent in some situations, separating environmental pollution from a vulnerability index maintains the ability to estimate the main effects of both pollution and vulnerability factors on the outcome. This allows for the identification of social and physical environmental features that may contribute to greater health impacts from pollution.

Our goal was to apply the ToxPi approach to construct an adaptable multidomain neighborhood environmental vulnerability index (NEVI) in an urban center. We aimed to characterize the overall magnitude of and identify patterns in neighborhood environmental vulnerability within a large, demographically and socioeconomically diverse, and densely-populated urban area. This index was compared to previously-used indices to characterize the additional information gained from using a multidomain index. Our proposed approach to account for racism exposure included index comparisons when constructed using downstream effects of racism rather than the composition of neighborhood-level racial/ethnic identities.

## Methods

### Study Area

We focused on New York City (NYC), an urban center with intra-urban variability in many social and structural features that can potentially contribute to environmental vulnerability [29]. To capture variation within NYC, we conceptualized our neighborhoods as census tracts, a relatively small spatial areal unit that could be aggregated into larger areas to correspond to other neighborhood definitions. In NYC, five distinct boroughs/counties (e.g. Bronx, Brooklyn, Manhattan, Queens, Staten Island) each have a current sociodemographic profile shaped by their unique development histories and informed by their respective municipal jurisdictions. Thus, to maximize the policy and public health relevance, we describe our NEVI use in the context of these five boroughs.

Of the 2,167 NYC census tracts, we excluded 51 tracts with populations less than twenty and 30 tracts with populations of at least twenty but missing data for at least one feature. Most of these 30 tracts were in non-residential areas, such as construction sites, parks, prisons, or universities. As a result, we ultimately included 2,086 census tracts in the NEVI. The Columbia University Institutional Review Board reviewed and approved the research protocol encompassing this analysis.

### Construction of Neighborhood Environmental Vulnerability Index

To construct the NEVI, we used data from the 2015–2019 U.S. Census American Community Survey (ACS) 5-year estimates and the 2020 Centers for Disease Control and Prevention (CDC) PLACES Project data release. ToxPi is a tool to integrate and visualize data across multiple domains [30]. Subject-matter knowledge is used throughout the overall index creation process, including the feature selection, grouping of the features into subdomains and domains, and weighting of all components. To select our domains, subdomains, and distinct area-level features, we conducted a literature search of research on social and structural drivers of vulnerability to environmental pollution [5, 6, 17, 19, 31–33]. Furthermore, we reviewed published vulnerability indices to compile and adapt domains from those applications of ToxPi for our purpose [10, 26–28, 34]. For example, the HGBEnviroScreen tool included a social vulnerability domain that we disaggregated into demographic and economic domains and three domains that measured environmental risks and exposures that we did not include to permit later potential effect modification analyses by those environmental measures [27]. Guided by the literature and research team’s subject-matter knowledge, we selected 4 primary domains composed of 24 subdomains and 54 distinct area-level features hypothesized to contribute to environmental vulnerability. The four primary domains were 1) demographics, 2) economic indicators (“economic”), 3) residential characteristics and density (“residential”), and 4) health behavior, outcomes, preventative practices, and access (“health status”) (Table 1). These four domains were equally weighted, so subdomains within each domain were weighted to achieve equal weighting at the domain level. Demographic, economic, and residential domain features were from the U.S. Census American Community Survey, while health status domain features were from the CDC PLACES Project (model-based estimates) [35, 36].

**Table 1:** Features Included in the Neighborhood Environmental Vulnerability Index

Domain^a^	Subdomain Represented by Slice (Number of Features, Weight of Overall Index)^a^	Features
Demographics (7 subdomains, 12 features)	Age (2 features, 1/28)	• Age under 18 (%) • Age 65 or older (%)
	Female-led household (1 feature, 1/28)	• Female-led households (%)
	Immigration (4 features, 1/28)	• Speak English "not at all" or "not well" (%) • Foreign-born (%) • Entered U.S. 2010 or later (%) • Not a U.S. citizen (%)
	Disability (1 feature, 1/28)	• With a disability (%)
	Single parent households (1 feature, 1/28)	• Children (age younger than 18) living with one parent (%)
	Transportation (2 features, 1/28)	• Who take public transportation, taxicab, bicycle, walking, or other non-automobile means (%) • Aggregate travel time to work
	Single households (1 feature, 1/28)	• Live alone (%)
Economic Indicators (6 subdomains, 7 features)	Income and poverty (2 features, 1/24)	• Household Income (median) • Income in past 12 months below poverty level (%)
	Occupation (1 feature, 1/24)	• Work in service, natural resources, construction, maintenance, production, transportation, or material moving occupations (%)
	Income inequality (1 feature, 1/24)	• Gini Index of income inequality
	Employment status (1 feature, 1/24)	• Unemployed among those in labor force aged 20–64 (%)
	Education (1 feature, 1/24)	• With less than high school education (%)
	Vehicle availability (1 feature, 1/24)	• With no vehicle available (%)
Residential Characteristics and Density (7 subdomains, 8 features)	Population density (1 feature, 1/28)	• Population per square mile (%)
	Group quarters (1 feature, 1/28)	• Live in group quarters (%); Group quarters include all locations not considered housing units. These include correctional facilities, nursing homes, mental hospitals, military barracks, group homes, missions, shelters, etc
	Household density (1 feature, 1/28)	• More than one occupant per room (%)
	Age of housing structure (1 feature, 1/28)	• Age of housing in 2019 (years)
	Units in housing structure (2 features, 1/28)	• Living in structure with 1 attached housing unit, 2 or more units, or in a mobile home, boat, RV, or van (%) • Living in structure with 20 or more units (%)
	Residential mobility (1 feature, 1/28)	• Moved in the past year (%)
	Housing vacancy (1 feature, 1/28)	• Vacancy (%)
Health Behaviors, Conditions, Prevention Practices, and Insurance Status (4 subdomains, 27 features)^b^	Health behaviors (4 features, 1/16)	• Currently smoke (%) • Binge drink (%) • Have no leisure-time physical activity (%) • Sleep for less than 7 h per day (%)
	Health conditions (14 features, 1/16)	• High blood pressure, 2017 (%) • High blood pressure medication use, among those with high blood pressure, 2017 (%) • Obesity (%) • Cancer, non-skin (%) • Current asthma (%) • Coronary heart disease (%) • Stroke (%) • COPD (%) • Diabetes (%) • High cholesterol, 2017 (%) • Chronic Kidney Disease (%) • Mental health not good for > = 14 days (%) • Physical health not good for > = 14 days (%) • All teeth lost (%)
	Prevention practices (8 features, 1/16)	• Routine checkup within past year (%) • Adult men aged 65 or older who are on date with clinical preventive services (flu shot past year, PPV shot ever, colorectal cancer screening) (%) • Adult women aged 65 or older who are on date with clinical preventive services (flu shot past year, PPV shot ever, colorectal cancer screening, mammogram within past 2 years) (%) • Visit to dentist or dental clinic (%) • Cervical cancer screening among women aged 21–65 (%) • Cholesterol screening, 2017 (%) • Colonoscopy screening among adults aged 50–75 years (%) • Mammography use among women aged 50–74 years (%)
	Health insurance status (1 feature, 1/16)	• Lack of health insurance (%)

**Data sources**: U.S. Census American Community Survey (ACS) 2015–2019 5-year estimates, Centers for Disease Control and Prevention (CDC) 2020 data release from the PLACES Project

^a^The four domains (demographics, economic indicators, residential characteristics and density, and health behaviors, conditions, prevention practices, and insurance status) were equally weighted, and each domain contained several subdomains. Subdomains were equally weighted *within* their domain, so any given subdomain was given a smaller overall weight if there were a greater number of other subdomains within its domain. For example, the household density subdomain was one of 7 subdomains within the economic indicators domain (1/4 weight), so its overall weight was 1/28 (= 1/4 * 1/7)

^b^Data for the Health Behaviors, Conditions, Prevention Practices, and Insurance Status domain were from the CDC PLACES Project, which provided prevalence estimates for health behaviors, health conditions, prevention practices, and health insurance status

The demographic domain consisted of 7 subdomains: age distribution, female-led households, nativity, disability, single parent households, transportation behaviors, and single households. For our primary analyses, we did not include racial and ethnic composition because systemic racism and related policies, rather than racial or ethnic identity, lead to greater social and economic stressors and environmental vulnerability [37]. Therefore, we only included stressors that reflect both individual-level aggregates and neighborhood-level characteristics. We conducted a sensitivity analysis evaluating if the NEVI varies when including racial and ethnic identity compositions. The economic domain included 6 subdomains: income and poverty status, occupation, income inequality, employment status, education, and vehicle availability. The residential domain included 7 subdomains: population density, group quarters, household density, age of housing structure, number of units in housing structure, residential mobility, and housing vacancy. Finally, the health status domain included 4 subdomains: health behaviors, health conditions, prevention practices, and health insurance status.

We used the ToxPi Graphical User Interface to calculate the overall NEVI and domain-specific scores, conceptualizing slices as subdomains within our four domains [38]. We first standardized each feature with z-scores because magnitudes varied greatly across features (e.g., median household income vs. percent below poverty level). Next, the software summed the values across the features within each subdomain before transforming those values by subtracting the minimum and dividing by the range of the values [39]. The resulting subdomain scores were then multiplied by the weights (specified in Table 1, with equally-weighted domains) to calculate the overall NEVI [39]. While the software calculates the overall NEVI and subdomain-specific scores, we manually calculated domain-specific scores by averaging the subdomain-specific scores within each of the four domains. ToxPi considers negative values as invalid or missing and ignores or converts negative values to zero (depending on if there are only negative values in a subdomain). Because of this, we re-centered features so that the minimum value would equal zero, resulting in values that were zero or greater across all features and the same original distribution (i.e., negative values not truncated). We coded features so that greater (i.e., more positive) values would indicate greater vulnerability. The final NEVI overall and domain-specific scores ranged from 0 to 1.

### Comparison to Existing Indices: NDI, SVI, EJI, and DAC

For comparison, we constructed an adapted Neighborhood Deprivation Index (NDI) originally developed by Messer et al. from the 2015-2019 U.S. Census ACS data and downloaded the Social Vulnerability Index (SVI) as originally prepared by CDC with tracts ranked within the state of New York (overall composite score only) [10]. In supplemental analyses, we compared NEVI with the CDC 2022 Environmental Justice Index (EJI) and NY State 2023 Disadvantaged Communities Score (DAC) [23, 40]. To construct a tract-level NDI that reflects heterogeneity across spatial strata of NYC, we adapted methods from both Messer et al. and Shmool et al. that we describe in the supplementary material [10, 14].

### Descriptive and Statistical Analyses

We calculated and described the distributions of the NEVI (overall, by domain, and by borough), NDI, and SVI. Next, we mapped both the overall index and domain-specific scores. To compare the NEVI to the NDI and SVI, we visualized quartile distributions of the indices in adjacent maps and calculated Spearman correlation coefficients between the NEVI and NDI/SVI across domain and borough. To identify common patterns in the NEVI subdomains across census tracts, we conducted a hierarchical clustering analysis with complete linkage and selected the optimal number of clusters using the Gap-statistic [41]. The composition of resulting clusters was compared using heat maps of the median subdomain scores, standardized by feature to compare relative scores. As a sensitivity analysis, all analyses were repeated after including racial and ethnic composition features within the NEVI demographic domain. Specifically, we included 4 features describing the proportion of residents who identified as Hispanic/Latino of any race, Black non-Hispanic/Latino, Asian non-Hispanic/Latino, and other non-White race non-Hispanic/Latino. Finally, we summarized overall and domain-specific vulnerability by high racial and ethnic composition per neighborhood, defined as having a racial and ethnic composition higher than the citywide median proportion. All data preprocessing and analyses were completed in R version 4.0.2. We used the *tidycensus* package to download U.S. Census data, [42] the *nycgeo* package to download NYC census tract shapefiles included in our choropleth maps, [43] and the *psych* package to perform PCA [44]. All programming code and data are available on Github jstingone/nevi.

## Results

### Overall NEVI and Domain-Specific Scores

**Fig. 1:**
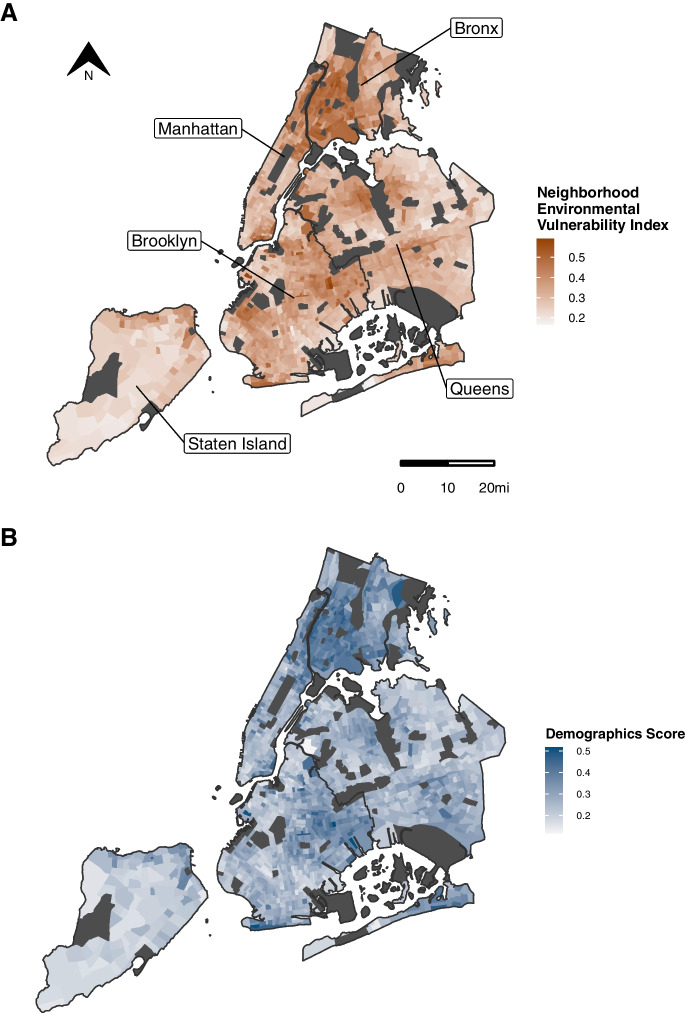

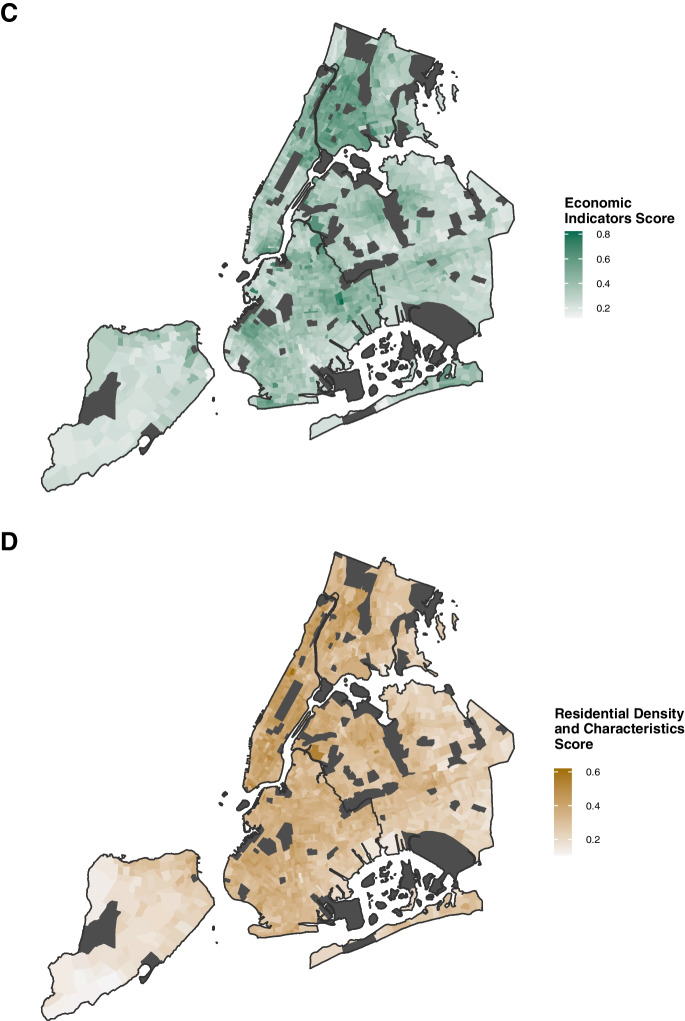

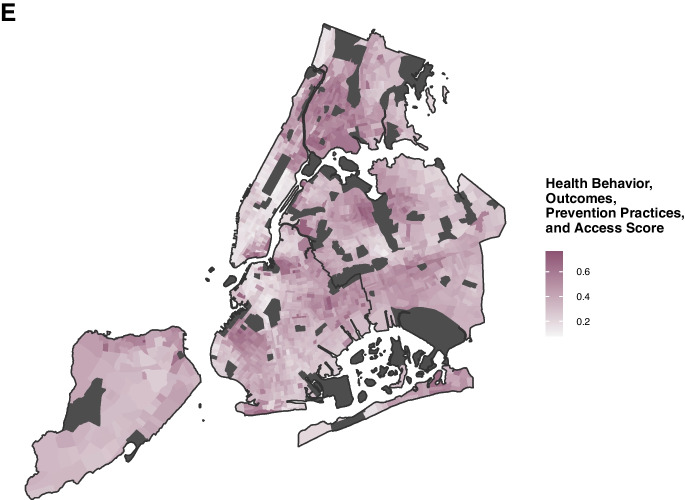
Maps of Neighborhood Environmental Vulnerability Index: Overall Index and Domain-Specific Scores Across New York City by Census Tract, 2015–2019. The maps display the distribution of the (**A**) overall Neighborhood Environmental Vulnerability Index across New York City along its (**B**-**E**) domain-specific scores. Areas that were excluded due to low population counts or missing features are shown in dark gray. Data Sources: U.S. Census American Community Survey 2015–2019 5-Year Estimates and Centers for Disease Control and Prevention PLACES Project 2020 Release

Figure 1 displays the distribution of the overall NEVI and domain-specific scores in separate maps. Supplemental Table S2 provides numeric estimates of the overall index and domain score distributions across census tracts.

As shown in Fig. 1A-D, the NEVI varied in overall magnitude and distribution of the domains across boroughs (Supplemental Table S2). The Bronx, an outer borough with mid-range population density and the lowest median income in NYC, had the highest median index (0.43, IQR = 0.35–0.48), while Staten Island, the borough with the lowest population-density and second highest median income, had the lowest median index (0.25, IQR = 0.23–0.30). Consistent with the overall index, median domain scores were the highest in the Bronx for the demographics (0.38, IQR = 0.32–0.41), economic (0.48, IQR = 0.37–0.57), and health status (0.49, IQR = 0.35–0.56) domains and the lowest in Staten Island for the demographics (0.21, IQR = 0.19–0.26), economic (0.25, IQR = 0.22–0.30), and residential (0.21, IQR = 0.18–0.25) domains (Supplemental Table S2, Supplemental Fig. S1). However, the median residential domain score was the highest (0.41, IQR = 0.37–0.44) and median health status domain score was the lowest (0.24, IQR = 0.16–0.42) in Manhattan, the borough with the highest population-density but also the highest median income.

### Comparisons Between NEVI, NDI, and SVI

**Fig. 2:**
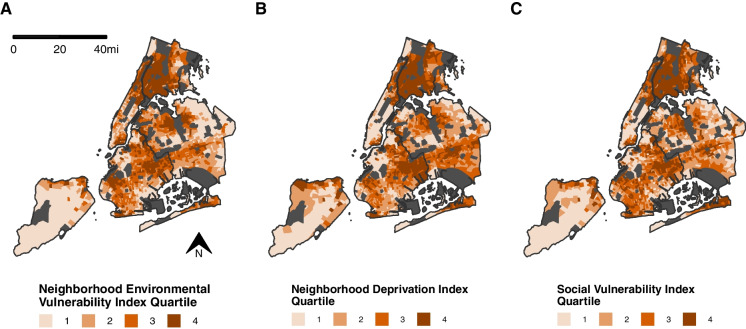
Maps of Neighborhood Environmental Vulnerability Index, Neighborhood Deprivation Index, and Social Vulnerability Index Across New York City by Census Tract, 2015–2019. The maps display the distribution of the (**A**) overall Neighborhood Environmental Vulnerability Index, (**B**) the Neighborhood Deprivation Index, and (**C**) the Social Vulnerability Index across New York City. Areas that were excluded due to low population counts or missing features are shown in dark gray. Data Sources: U.S. Census American Community Survey 2015–2019 5-Year Estimates, Centers for Disease Control and Prevention PLACES Project 2020 Release, and Centers for Disease Control and Prevention/Agency for Toxic Substances and Disease Registry 2018 Social Vulnerability Index

Overall, there were strong, positive correlations between NEVI and NDI (r = 0.91, *p* < 0.001) and NEVI with SVI (r = 0.87, *p* < 0.001). However, there were some notable distribution differences with visual comparisons by borough, including less agreement between the indices in Queens, the most ethnically-diverse borough [45] (Fig. 2). Correlations between the overall NEVI and NDI/SDI were lower in Queens than other boroughs, especially for the NDI (Supplemental Fig. S2). The NDI explained less variance in Queens than other boroughs. Correlations between the domain-specific scores and the NDI and SVI were high, except between the NEVI residential score which consistently showed lower correlations with both the NDI and the SVI across boroughs. In supplemental analyses, we saw similar lower correlations between the NEVI residential score with the EJI and DAC (Supplemental Fig. 5A, B, and C).

### Vulnerability Profiles of Census Tracts

**Fig. 3:**
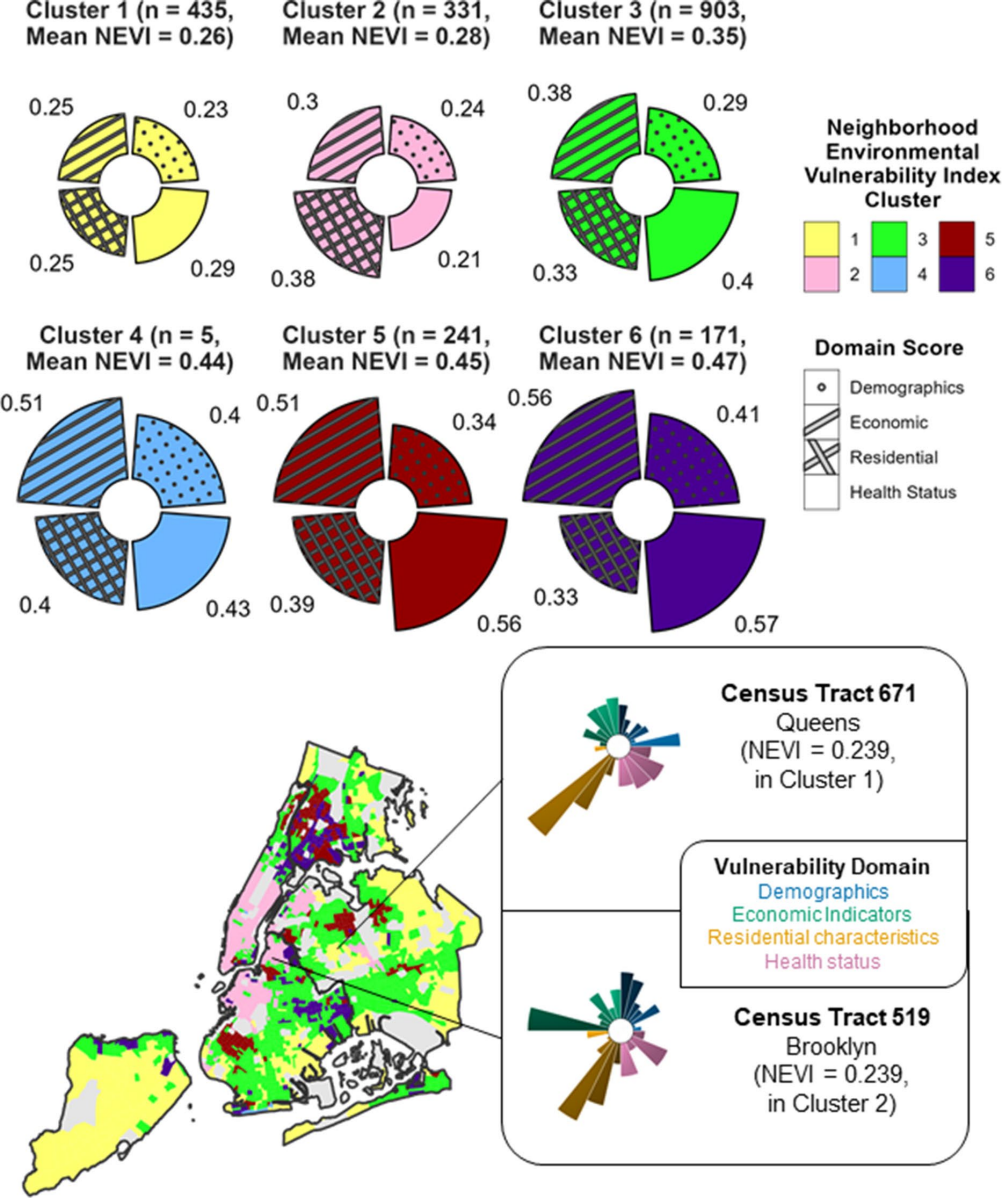
Map of Clusters with Example Tract Vulnerability Profiles for the Neighborhood Environmental Vulnerability Index across New York City, 2015–2019. The vulnerability profiles for the six clusters (on top) and two census tracts are shown: Census Tract 671 in Queens and Census Tract 519 in Brooklyn (on bottom right). The different colors represent different clusters in the cluster vulnerability profiles, and the different colors in the Census tract vulnerability profiles represent different domains. Within each domain of the Census tract vulnerability profiles, the different shades of each slice represent various subdomains, with larger slices representing greater vulnerability. The map (bottom left) shows the distribution of the NEVI clusters across NYC, with areas that were excluded due to low population counts or missing features shown in dark gray. Data Sources: U.S. Census American Community Survey 2015–2019 5-Year Estimates and Centers for Disease Control and Prevention PLACES Project 2020 Release

As an example of how domain-specific features contribute differently to NEVI and support a targeted and adaptable public health approach, in Fig. 3, we show vulnerability profiles for two sample NYC census tracts with similar overall NEVI scores (0.239): Census Tract 671 and Census Tract 519. Despite having similar overall scores, the patterns across subdomain scores are different with greater economic vulnerability in Census Tract 519 (0.64 vs 0.23 economic sub-score) and slightly greater scores in residential and health-related features in Census Tract 671.

### Vulnerability Profiles of NEVI Clusters

**Fig. 4:**
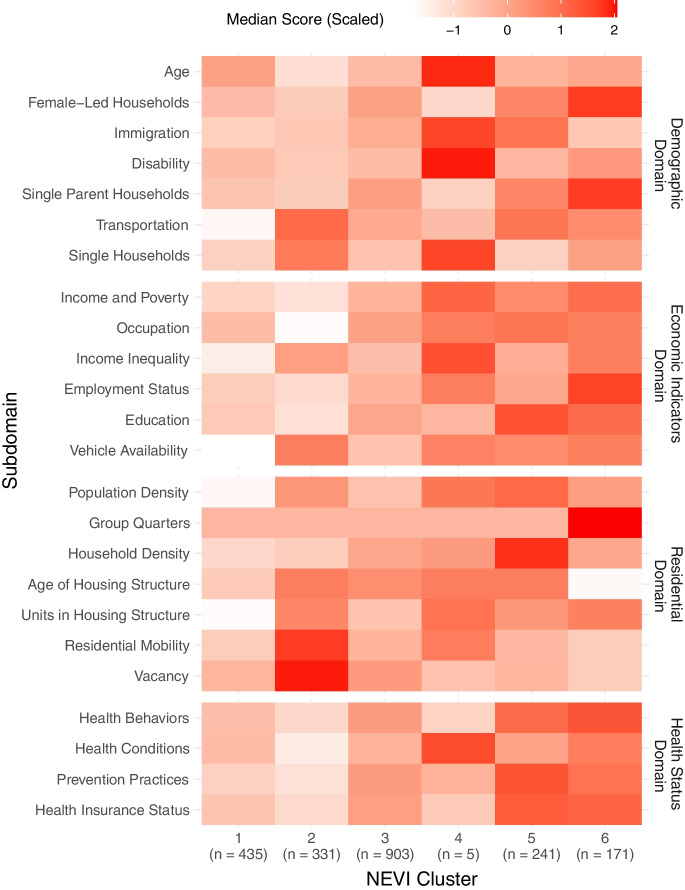
Heatmap of Median Subdomain Scores by Neighborhood Environmental Vulnerability Index Cluster. The median subdomain scores were standardized by feature to emphasize the relative magnitude of the subdomain scores across clusters

Our clustering analysis revealed 6 patterns in NEVI domain scores across census tracts (Fig. 3) — two low, one medium, and three high vulnerability clusters: Cluster 1) low across all domains (median NEVI = 0.26, n = 435); Cluster 2) primarily low but greater residential and very low health vulnerability (median NEVI = 0.28, n = 331); Cluster 3) medium vulnerability across all domains (median NEVI = 0.35, n = 903); Cluster 4) high vulnerability across demographic, residential and economic domains (median NEVI = 0.44, n = 5); Cluster 5) high vulnerability across economic, residential and health status domains (median NEVI = 0.45, n = 241); and Cluster 6) high vulnerability across demographic, economic and health status domains (median NEVI = 0.47, n = 171). We created a heatmap (Fig. 4) to visualize subdomain differences across clusters. For example, the low vulnerability Cluster 2 had consistently lower health status vulnerability scores than Cluster 1 but had higher scores in age of and units in housing structure, residential mobility, and vacancy within the residential domain. Additionally, the high vulnerability Cluster 6 consistently had lower residential vulnerability than the other high vulnerability clusters, except for the location of group quarters but higher vulnerability related to employment status and single-parent or female-led households.

### Sensitivity Analyses by Race and Ethnicity

The NEVI did not change considerably when including racial and ethnic composition. Supplemental Fig. S3 illustrates that the overall NEVI mean values and distributions remained similar in NYC overall or by borough when including racial and ethnic composition. The demographic domain scores changed slightly, particularly in Queens where inclusion of race/ethnicity features slightly increased mean domain scores. Cluster patterns changed slightly when including race/ethnicity features in the NEVI (Supplemental Fig. S4). For example, some census tracts were regrouped from medium to low vulnerability clusters, leading to more low-vulnerability areas. Furthermore, some census tracts were reclassified from high to medium vulnerability clusters. This caused small changes in the overall cluster vulnerability scores, but most census tracts remained within the same cluster.

**Fig. 5:**
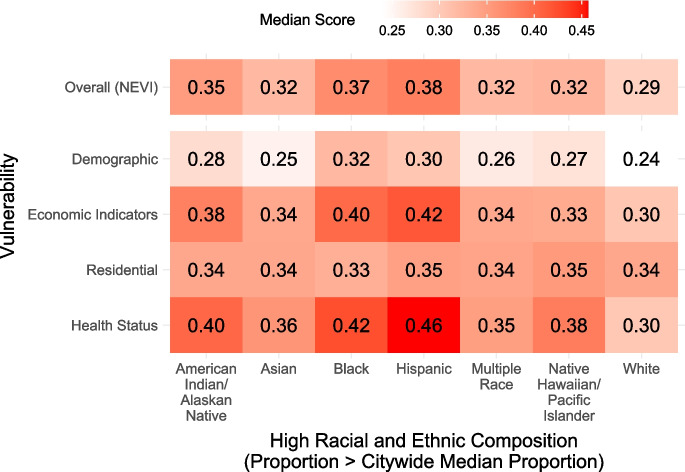
Median Overall and Domain Scores by High Racial and Ethnic Composition. The median NEVI and domain-specific scores are shown by high racial and ethnic composition across neighborhoods, defined as having a racial and ethnic composition higher than the citywide median proportion. A given neighborhood may have a high composition across multiple racial and ethnic groups (e.g., a neighborhood with both 1) % Hispanic higher than the citywide median % Hispanic and 2) % Native Hawaiian/Pacific Islander higher than the citywide median % Native Hawaiian/Pacific Islander)

When comparing vulnerability across race and ethnicity, overall vulnerability was lowest in White (neighborhood composition higher than the citywide median proportion) neighborhoods (median NEVI = 0.29), moderate in Asian, Multiple Race, and Native Hawaiian/Pacific Islander neighborhoods (median NEVI = 0.32), and highest in American Indian/Alaskan Native, Black, and Hispanic neighborhoods (median NEVI = 0.35, 0.37, and 0.38, respectively) (Fig. 5). When disaggregating by domain, residential vulnerability was similar across racial and ethnic groups, and economic and health status vulnerability was especially high among American Indian/Alaskan Native, Black, and Hispanic neighborhoods.

## Discussion

The NEVI characterized neighborhood environmental vulnerability across NYC and quantified contributions to vulnerability across four domains: 1) demographics, 2) economic indicators, 3) residential characteristics, and 4) health status. There was general agreement between the overall NEVI and previously developed indices for deprivation (NDI) and social vulnerability (SVI). However, the NEVI offered additional benefits, including its adaptable construction and additional information about the types of vulnerability across a geographic area. Together, these can inform efforts to enhance subsequent environmental justice research and interventions aimed at reducing vulnerability to environmental pollutant exposures in neighborhoods at the hyperlocal scale.

Our index characterized potential vulnerability contributions from different domains, compared to previous indices that only provide an overall score for vulnerability or deprivation [10, 24]. Prior studies including a single deprivation measure had limited ability to describe which neighborhood environmental aspect were related to their results [46, 47]. Knowing which domains contribute to higher neighborhood vulnerabilities would facilitate adaptable population health planning and research to allocate certain types of resources to targeted neighborhoods even if the domain or specific features are not themselves modifiable, such as tailoring interventions to specific vulnerable demographic groups in a given neighborhood. A recent study compared two models with different covariates (a heat vulnerability index or a multivariable approach with individual features as predictors of heat-related mortality) and found that the multivariable approach resulted in a better model fit [48]. The study results highlight a trade-off between simplifying constructs of interest (i.e., using one overall index) and losing information from combining individual features. Our approach retains some of the information lost from summarizing vulnerability as an overall score and provides theoretically useful vulnerability subcategorizations by offering domain-specific scores.

Examining patterns across domains also provides greater information than a single score. Our clustering analysis revealed six primary patterns of vulnerability, which differentiated boroughs in more detail than conveyed by the absolute magnitude. For example, clusters 1 and 2 both similarly had low overall vulnerability. However, vulnerability in cluster 2 was lower due to existing health conditions but greater due to residential characteristics. This could point to different routes of intervention to reduce and/or ameliorate related health impacts of environmental pollution. For example, when designing an intervention to reduce air pollution health effects, one may target better chronic disease management in Cluster 1 while focusing on improved ventilation in the older housing stock of Cluster 2.

Notably, we excluded racial and ethnic composition measures in the final index construction. Communities of color disproportionately experience worse health outcomes resulting from environmental pollutant exposure and greater amounts of social and economic stressors across the US. For example, racial segregation and historic racist real estate practices such as redlining led to concentrated poverty and structural disinvestment in racially minoritized urban neighborhoods that could increase their vulnerability to the health impacts of environmental pollutants [32, 33, 49–52]. Supported by prior evidence, all economic, residential, and health status subdomain features, as well as some demographic subdomain features (e.g., transportation), were identified as downstream impacts of structural and systemic racism. Therefore, rather than using neighborhood-level racial and ethnic identity composition, these subdomain features were operationalized as proxy measures of exposure to racism. Our sensitivity analyses revealed that there was little difference in overall NEVI scores when including racial and ethnic identity compositions. This is likely due to socioeconomic, residential and health-related outcomes being highly correlated with race through the impacts of racism. A previous analysis of the Climate and Economic Justice Screening Tool, developed by the White House, also found similarity in community rankings regardless of whether racial and ethnic demographics were included [53]. However, other contexts seeking to use an index like the NEVI may want to include racial and ethnic identity compositions as proxies for other, unmeasured indicators of structural racism. This separation of race and ethnic composition from NEVI allowed us not only to identify higher overall vulnerability in communities of color but also that the vulnerability variations were driven by economic and health status differences, reflective of US racial and ethnic disparities. We recommend that future studies further validate the NEVI across other exposures and in causal analyses with health outcomes.

The NEVI and NDI/SVI were generally similar across boroughs and domains, except the NEVI residential domain (i.e., lower correlations), likely because no or fewer housing-related features linked to environmental hazards were included in the NDI/SVI [5, 54, 55]. Overall, NEVI had a lower correlation with NDI/SVI in Queens and the NDI explained less total variation in Queens than other NYC boroughs, possibly because Queens is the most demographically and socioeconomically diverse borough in NYC [45, 56]. One limitation of the NEVI stemmed from our need to exclude some census tracts due to low population or missing features. This limits its potential applicability in “non-residential” areas, such as prisons, with possibly greater environmental vulnerability [57, 58]. Additionally, we currently only used the NEVI to describe vulnerability in urban centers, potentially limiting the generalizability of our observed vulnerability patterns to suburban and rural areas. However, the adaptable NEVI creation process would facilitate index creation in other areas to ascertain their distinct vulnerability patterns.

The NEVI provides several advantages that increases its interpretability and utility. First, we were able to characterize the magnitude of potential environmental vulnerability, quantify the contributions from various domains, and identify vulnerability patterns across an urban area. These provide additional interpretability that could better inform public health planning and additional domain-specific scores to evaluate in research studies incorporating vulnerability measures. Next, ToxPi can be used with a graphical user interface or an R package, promoting greater usability. Furthermore, ToxPi enables background knowledge use to inform feature selection and weighting approaches, unlike indices that apply purely data-driven methods to retain features and may drop theoretically important features highly correlated with other features. Being able to choose features and specify weights for vulnerability contributions allows for a hybrid approach in which more hypothesis-driven information may be incorporated in index construction. The NEVI customization process (choosing features and weights) to fit specific hypotheses is transparent, facilitating discussion and critique. For example, we selected four domains that were hypothesized to reflect distinct area-level characteristics contributing to environmental vulnerability, distinct from the environmental pollution concentrations that other indices might include to identify areas most at-risk of specific health outcomes. Compared to the SVI or other indices that includes pre-set domains, using ToxPi enables adding domains and/or features in a clear manner, that promotes flexibility for the investigator and transparency for communities and stakeholders seeking to interpret the vulnerability index.

## Conclusion

We developed a neighborhood-level index to measure vulnerability to health impacts from environmental pollution for New York City and found that our NEVI was generally consistent with previously-developed deprivation and vulnerability scores. However, the NEVI was additionally able to characterize contributions to vulnerability across multiple domains, providing greater insight into intra-urban variation in vulnerability. Specifically, the customization option of this index-building approach allowed theory-based analysis of specific features/domain contribution to the index score (as we explored with racial composition). This metric can be used to inform targeted public and environmental health research and practice at the hyperlocal scale and improve our understanding of the impact of environmental exposures on communities with varying levels of vulnerability.

### Supplementary Information

Below is the link to the electronic supplementary material.

Supplementary file1 (DOCX 3391 KB)

## Data Availability

The data used in this study are are available on Github (github.com/jstingone/nevi).
